# Role of HER-2/Neu Expression in Premalignant and Malignant Lesions of the Uterine Cervix: A Pathological Study

**DOI:** 10.7759/cureus.78211

**Published:** 2025-01-29

**Authors:** Joyeeta Mandal, Anshu Jamaiyar, Sona Pathak

**Affiliations:** 1 Pathology, Rajendra Institute of Medical Sciences, Ranchi, IND

**Keywords:** her-2-neu, her-2 neu protein, immunohistochemistry staining, oncopathology, uterine cervical cancer

## Abstract

Background: HER-2/neu, a membrane-bound receptor encoded on chromosome 17q21, is involved in carcinogenesis across several cancers, including breast, stomach, and cervical cancer. This study evaluates HER-2/neu expression in premalignant and malignant cervical lesions and correlates it with histological types and grades.

Methods: This prospective hospital-based study, conducted at the Department of Pathology of a tertiary care teaching hospital in Eastern India, included 74 cases (11 premalignant, 63 malignant) from January 2023 to October 2024. HER-2/neu expression was analyzed using immunohistochemistry (IHC), and scoring was based on the American Society of Clinical Oncology-College of American Pathologists (ASCO-CAP) 2013 guidelines. Statistical analysis was performed to determine correlations.

Results: HER-2/neu positivity was observed in 8 (10.8%) out of 74 cases. Among squamous cell carcinomas (SCC), poorly differentiated cases showed maximum positivity in 4 (66.7%) cases, followed by moderately differentiated SCC in 2 (33.3%) cases, with no positivity in well-differentiated SCC. Among adenocarcinomas (a total of 10 (13.5%) among 74 in number), only 1 (12.5%) out of 8 cases were found to be Her-2/neu positive. Premalignant lesions demonstrated positivity in one (9.1%) case only. There was no statistically significant correlation between HER-2/neu expression and histological grade or presence of malignancy.

Conclusions: HER-2/neu expression is higher in malignant cervical lesions compared to premalignant ones, suggesting its role in tumor progression. Further studies with larger sample sizes and additional variables like human papillomavirus (HPV) status are recommended to explore its prognostic potential.

## Introduction

Cervical cancer remains a major global health concern, ranking as the fourth most common cancer in women. In 2020, an estimated 604,000 new cases and 342,000 deaths were reported worldwide [1]. Although early screening has significantly reduced cervical cancer-related mortality in developed nations, the disease burden remains high in less-developed regions where late-stage diagnosis is common.

Receptors and molecular markers play a crucial role in the pathogenesis of cervical carcinomas and pre-malignant lesions. Markers such as human papillomavirus (HPV) DNA, p16INK4a, and Ki-67 are commonly used to assess tumor progression and predict clinical outcomes. The etiological role of high-risk HPVs, particularly HPV-16 and HPV-18, is well-documented [2]. While HPV infection alone is insufficient for malignant transformation, host and viral factors such as genetic instability and oncoprotein activity play pivotal roles. The overexpression of p16INK4a, for instance, indicates HPV-related oncogenic activity and is often associated with high-grade lesions [3]. Similarly, alterations in markers like epidermal growth factor receptor (EGFR), vascular endothelial growth factor (VEGF), and molecular pathways involved in cell cycle regulation contribute to tumor growth and metastasis [4]. HER-2/neu, also called c-erbB-2, stands for “human epidermal growth factor receptor 2”, and is a cell membrane-bound receptor localized on chromosome 17q21 that encodes a growth factor-like molecule with tyrosine kinase activity [5]. It is one such oncogene implicated in carcinogenesis, with overexpression associated with poor prognosis, aggressive tumor behavior, and metastatic potential in various cancers, including cervical cancer [6].

Many studies have convincingly shown that regression of c-erbB-2 suppresses the malignant phenotypes of cancer cells overexpressing this oncoprotein, which may serve as an excellent target for developing anti-cancer agents specific for c-erbB-2 overexpression [7]. This immunohistochemistry (IHC) study investigates the expression of HER-2/neu in premalignant and malignant cervical lesions and its association with histological subtypes and tumor grades, aiming to contribute to the understanding of its potential prognostic value.

This study aims to establish the patterns of HER-2/neu expression in premalignant and malignant cervical lesions and correlate HER-2/neu expression patterns with histological types and grades of cervical tumors.

## Materials and methods

This hospital-based prospective study was conducted in the pathology department of the Rajendra Institute of Medical Sciences in Eastern India from January 2023 to October 2024.

Sample collection

The study included cervical biopsies and hysterectomy specimens from 74 patients aged 20-78 years. Inclusion criteria encompassed histologically confirmed premalignant or malignant cervical lesions. Cases diagnosed with inflammatory or benign conditions or lacking adequate tissue for analysis were excluded.

Tissue processing and IHC protocol

**Figure 1:**
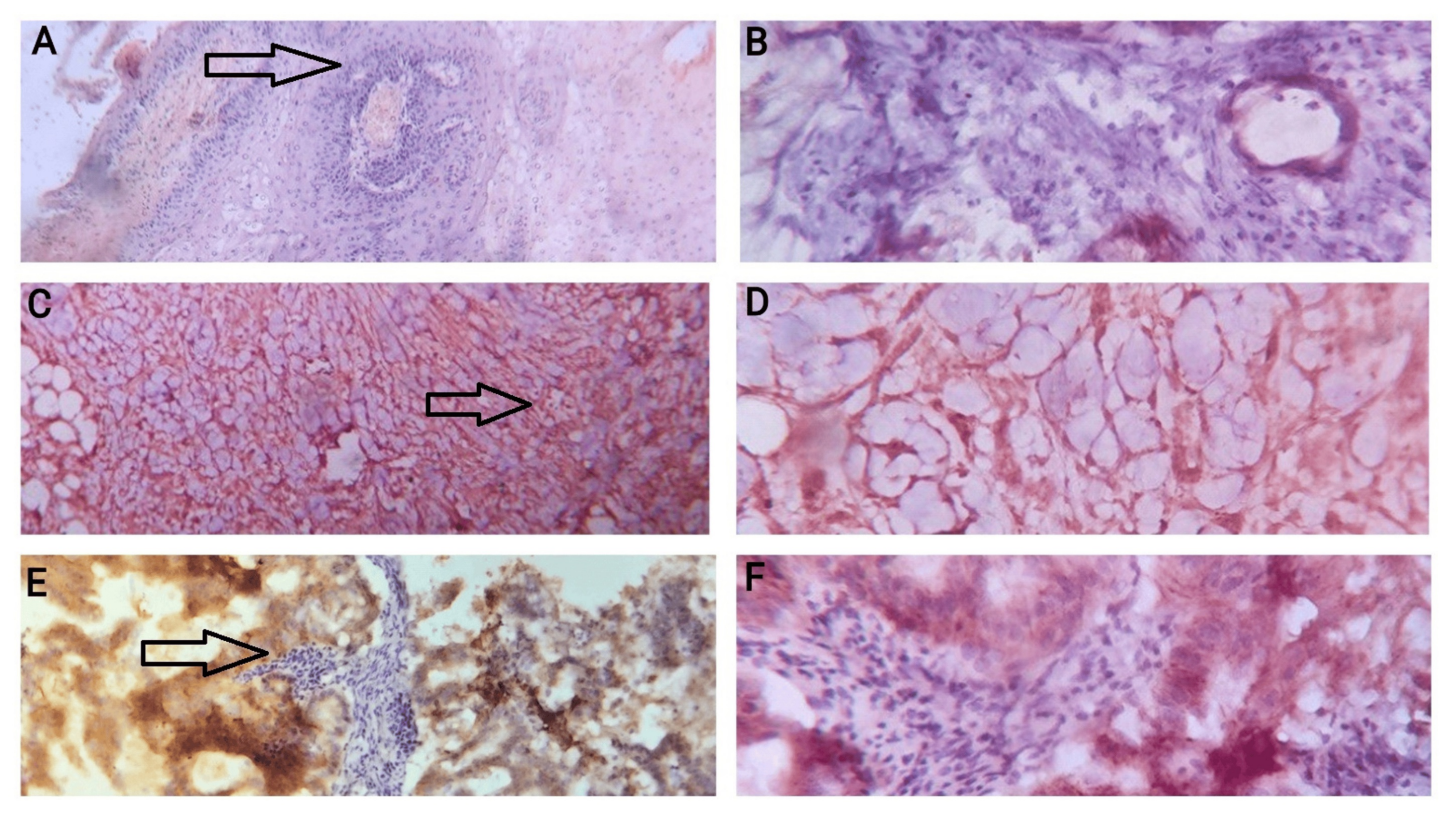
Photomicrographs of IHC-stained slides of cervical carcinomas included in the study A: Photomicrograph of an SCC of the cervix from the study showing 1+ HER-2/neu staining in 10x view (the black-bordered arrow-pointed area has been zoomed in Part B). B: Photomicrograph of an SCC of the cervix from the study showing 1+ HER-2/neu staining in 40x view. C: Photomicrograph of an SCC of the cervix from the study showing 3+ HER-2/neu staining in 10x view (the black-bordered arrow-pointed area has been zoomed in Part D). D: Photomicrograph of an SCC of the cervix from the study showing 3+ HER-2/neu staining in 40x view. E: Photomicrograph of the adenocarcinoma of the cervix from the study that showed 3+ HER-2/neu staining in 10x view (the black-bordered arrow-pointed area has been zoomed in Part F). F: Photomicrograph of the adenocarcinoma of the cervix from the study that showed 3+ HER-2/neu staining in 40x view. IHC: immunohistochemistry; SCC: squamous cell carcinomas

Fixation was done with 10% buffered formalin. Dehydration was carried out by isopropyl alcohol in graded concentrations. Clearing and embedding were done in xylene and paraffin wax. Sectioning was done into 5 µm thick sections mounted on poly-L-lysine-coated slides. Staining was done by hematoxylin and eosin (H&E) for histological diagnosis and HER-2/neu IHC using indirect immunoperoxidase techniques (Proprietary Vitro Master Diagnostica kit; 1:10 dilution factor; Catalogue no. MAD-000308QD-R; Lot no. 03080022). HER-2/neu staining was graded following the American Society of Clinical Oncology-College of American Pathologists (ASCO-CAP) 2013 guidelines (Figure 1) [8].

Statistical analysis

Descriptive statistics were performed. Proportions and frequencies were mentioned for categorical variables while the median with standard deviation was used for continuous variables. Data were analyzed using chi-square tests to determine correlations between HER-2/neu expression and clinicopathological variables. A p-value of less than 0.05 was considered significant. All statistical analysis was carried out by proprietary IBM SPSS Statistics for Windows, Version 25 (Released 2017; IBM Corp., Armonk, New York, United States).

## Results

Among the 74 cases analyzed, the maximum number of patients belonged to the age group of 41-50 years (32.4%). A +1 HER-2/neu score was deemed negative; a +3 HER-2/neu score was deemed positive. There were no +2/equivocally stained cases in the study. HER-2/neu positivity was noted predominantly in the age groups 31-40 years and 41-50 years (37.5% each). The majority of cases, i.e., 63 (85.1%), were malignant lesions, with 53 (71.6%) being squamous cell carcinoma (SCC) and 10 (13.5%) adenocarcinoma. Premalignant lesions (low-grade squamous intraepithelial lesion (LSIL) and high-grade squamous intraepithelial lesion (HSIL)) constituted 11 (14.9%) cases. Among 63 cases of carcinoma cervix, HER-2/neu expression is noticed in seven cases (11.1%). Out of these seven cases, six cases (85.7%) are SCC and one case (14.3%) is adenocarcinoma. One case of HER-2/neu positivity (9.1%) was found among the 11 premalignant lesions (LSIL). Among SCC, poorly differentiated cases exhibited the highest positivity in four (66.7%) cases, followed by moderately differentiated SCC in two (33.3%) cases. No well-differentiated SCC cases showed HER-2/neu expression. Premalignant lesions demonstrated HER-2/neu positivity in one (19.1%) case. Hence, in the clinicopathological sense, poorly differentiated cervical SCC had the highest incidence of HER-2/neu expression. The age-wise and histopathological distribution with the respective HER-2 positivity rates has been enumerated in Table 1.

**Table 1:** Age and histopathological distribution of study cases The frequencies have been represented in N (%) format. For the chi-square test, degrees of freedom = 5, chi-square number = 2.697, p = 0.074 LSIL: low-grade squamous intraepithelial lesion; HSIL: high-grade squamous intraepithelial lesion; SCC: squamous cell carcinoma

Age group (in years)	No. of cases (%); n = 74	No. of HER-2 positive cases (%); n = 8 out of 74
21-30	4 (5.4%)	0
31-40	13 (17.6%)	3 (37.5%)
41-50	24 (32.4%)	3 (37.5%)
51-60	19 (25.7%)	1 (12.5%)
61-70	11 (14.9%)	0
71-80	2 (2.7%)	0
81-90	1 (1.4%)	1 (12.5%)
Histopathological type	No. of cases (%); n = 74	No. of HER-2 positive cases (%); n = 8 out of 74
LSIL	7 (9.5%)	1 (12.5%)
HSIL	4 (5.4%)	0
Well differentiated SCC	12 (16.2%)	0
Moderately differentiated SCC	15 (20.3%)	2 (25%)
Poorly differentiated SCC	26 (35.1%)	4 (50%)
Adenocarcinoma	10 (13.5%)	1 (12.5%)
Pearson’s chi-square test	χ^2^ (5, N = 74) = 2.697	p = 0.074

However, statistical analysis by Pearson’s chi-square test revealed no statistically significant correlation between HER-2/neu expression and histological grade; χ^2^ (5, N = 74) = 2.697, p = 0.074 (Table 1).

Also, there was no statistically significant correlation between HER-2/neu expression and the presence of malignancy in the tissue; χ^2^ (1, N = 74) = 0.4, p = 0.661 (Table 2).

**Table 2:** Cross tabulation between incidence of malignant or premalignant lesions with HER-2/neu positivity rates The frequencies have been represented in N (%) format. For the chi-square test, degrees of freedom = 1, chi-square number = 0.4, p = 0.661

Presence of malignancy	No. of cases (%); n = 74	No. of HER-2 positive cases (%); n = 8 out of 74
Pre-malignant	11 (14.9%)	1 (12.5%)
Malignant	63 (85.1%)	7 (87.5%)
Pearson’s chi-square test	χ^2^ (1, N = 74) = 0.4	p = 0.661

## Discussion

The role of HER-2/neu, a membrane-bound receptor involved in cell proliferation and differentiation, in cervical carcinoma has been a subject of significant interest. In the present study comprising 11 premalignant and 63 malignant lesions, SCC cases 53 (71.6%) by count outnumbered the adenocarcinoma cases 10 (13.5%) by count, which was in concordance with the World Health Organization (WHO), which states that SCCs account for ∼70%-80% of cervical cancers while adenocarcinomas for 10%-15% [9].

This study evaluated HER-2/neu expression in premalignant and malignant cervical lesions, revealing an overall positivity rate of 8 (10.8%) out of 74 cases, with a notable association with poorly differentiated SCC. With the review of the literature available, it was observed that the studies designed to examine HER-2/neu in lesions of the uterine cervix showed inconsistent results wherein HER-2/neu positive staining in invasive carcinoma cervix ranged from 14% to 100% in various studies [10-12].

HER-2/neu expression was found predominantly in malignant lesions compared to premalignant ones. Among malignant lesions, poorly differentiated SCC exhibited the highest expression in four (66.7%) cases, followed by moderately differentiated SCC in two (33.3%) cases, with no expression observed in well-differentiated SCC. This suggests a potential correlation between HER-2/neu expression and the degree of tumor differentiation, reflecting its role in aggressive tumor behavior and poor prognosis.

These findings align with those reported by Patel et al., who documented HER-2/neu positivity in 11.53% of cervical lesions. In their study, HER-2/neu positivity expression was observed in 50% of cases of SCCs, 20% of poorly differentiated carcinomas, 18.1% of well-differentiated carcinomas, and 3.4% of moderately differentiated carcinomas [13].

Gupta et al. also observed a similar significant HER-2/neu expression in carcinoma cervix, reporting a positivity rate of 63%, with poorly differentiated SCC showing a higher prevalence of HER-2/neu positivity of 80%​ [14].

The variability in HER-2/neu positivity across studies is noteworthy, with rates ranging from <1% (Fadare and Zheng) [15] to 63% (Gupta et al.)​ [10]. Several factors may contribute to these discrepancies, including differences in sample size, scoring criteria, and antigen retrieval methods.

The present study's positivity rate of 8 (10.8%) out of 74 cases closely mirrors that reported by Patel et al. (11.53%) and Bajpai et al. (35.7%) in their analysis of premalignant and malignant cervical lesions​ [13,16]. These results underscore the heterogeneity in HER-2/neu expression in cervical cancers and its dependence on tumor type, grade, and methodology.

Expression of HER-2/neu among malignant cases was found to be higher in our study as compared to premalignant lesions, though not statistically significant, which is similar to the findings of Bajpai et al. and Patel et al. [13,16]. This indicates the possibility of a gradual progression of HER-2/neu expression from premalignant to malignant lesions of the cervix.

The findings of HER-2/neu expression in grades of SCC closely mimicked those in the study done by Gupta et al. [14]. The comparison of HER-2/neu positivity rates in different studies with that of our study has been demonstrated in Table 3.

**Table 3:** Comparison of percentages of HER-2/neu expression among different studies Only percentages of findings in the mentioned studies have been tabulated as exact frequency counts are beyond the scope of this discussion.

Name of study (in chronological order)	HER-2/neu expression rates
Nakano et al. (1994) [8]	43%
Fadare and Zheng (2004) [11]	<1%
Gupta et al. (2009) [10]	63%
Bajpai et al. (2017) [12]	35.7%
Patel et al. (2018) [9]	11.5%
Present study	10.8%

The role of HER-2/neu as a prognostic marker in cervical carcinoma remains inconclusive. While its overexpression has been correlated with aggressive tumor behavior and poor prognosis in breast and ovarian cancers, its prognostic significance in cervical carcinoma is less clear. Nakano et al. [12] demonstrated that HER-2/neu expression was an independent prognostic factor in advanced cervical carcinoma, with a positivity rate of 43%. Conversely, the lack of significant correlation between HER-2/neu expression and tumor grade in the present study (p = 0.747) suggests that additional factors, such as HPV status and molecular alterations, may modulate its expression and clinical implications. Its potential as a therapeutic target in cervical cancer warrants further investigation, particularly in advanced or refractory cases. Bajpai et al. [16] highlighted the feasibility of monoclonal antibody-based therapies for HER-2/neu-positive cervical cancers, emphasizing the need for standardized testing protocols to identify eligible patients. Additionally, further exploration of Her-2/neu 2+ cases (though there were none in the present study) should be done in a larger study and should be run in high-volume centers, in which fluorescent in situ hybridization (FISH) facilities are available, as there is an upcoming concept of "low Her-2/neu" tumors under research.

Strengths and limitations

This study adds to the growing body of literature on HER-2/neu expression in cervical lesions, providing valuable insights into its prevalence and association with histological subtypes. However, the relatively small sample size (n = 74) and lack of data on clinical stage, lymph node status, and HPV subtype represent significant limitations. Future studies incorporating these parameters and larger cohorts are essential to validate the findings and clarify the prognostic and therapeutic implications of HER-2/neu expression in cervical carcinoma.

Future directions

Given the heterogeneous expression of HER-2/neu in cervical carcinoma, a multidisciplinary approach integrating molecular diagnostics, histopathological evaluation, and clinical staging is crucial for accurate prognostication and treatment planning. Further research should focus on evaluating HER-2/neu expression in relation to HPV genotype and viral load, assessing the efficacy of HER-2/neu-targeted therapies in cervical carcinoma, particularly in advanced and recurrent cases, and exploring the molecular mechanisms underlying HER-2/neu overexpression in cervical cancer and its interplay with other oncogenic pathways.

## Conclusions

This study investigates the involvement of HER-2/neu in the development of cervical cancer, revealing a positivity rate of 10.8%, primarily observed in poorly differentiated SCCs. The research suggests that the presence of HER-2/neu is linked to more aggressive tumor behavior, highlighting its potential as a biomarker for tracking disease progression. Although HER-2/neu has been well-established as a therapeutic target in other cancer types, its clinical relevance in cervical cancer remains under-explored. Interestingly, the study found no significant correlation between HER-2/neu expression and tumor grade, indicating the complexity of its role in cervical carcinogenesis and signaling the need for deeper investigation to fully understand its implications.

Looking ahead, future studies with larger sample sizes and a broader scope - including the integration of clinical parameters, a closer look at HER-2/neu 2+ cases, and the use of more advanced diagnostic tools like FISH - are essential for confirming these initial findings. Additionally, incorporating HPV status into future research could provide more comprehensive insights into the relationship between HER-2/neu and cervical cancer. Targeted HER-2/neu therapies may also offer promising new treatments, especially for patients with advanced or refractory cases. In conclusion, while HER-2/neu shows considerable promise as both a diagnostic and therapeutic target, ongoing and expansive research is crucial to unlocking its full potential in improving patient outcomes in cervical carcinoma.
